# Real-Time Pig Weight Assessment and Carbon Footprint Monitoring Based on Computer Vision

**DOI:** 10.3390/ani15172611

**Published:** 2025-09-05

**Authors:** Min Chen, Haopu Li, Zhidong Zhang, Ruixian Ren, Zhijiang Wang, Junnan Feng, Riliang Cao, Guangying Hu, Zhenyu Liu

**Affiliations:** 1 Department of Big Data and Intelligent Engineering, Shanxi Institute of Technology, Yangquan 045000, China; cmlhp0612@163.com (M.C.); zhaozhid5220@yeah.net (Z.Z.); renruixian@sxit.edu.cn (R.R.); 2 College of Agricultural Engineering, Shanxi Agricultural University, Taigu, Jinzhong 030801, China; lihp0307@163.com (H.L.); 13453846074@163.com (J.F.); 3 College of Information Science and Engineering, Shanxi Agricultural University, Taigu, Jinzhong 030801, China; wangjw9211@163.com; 4 College of Animal Science, Shanxi Agricultural University, Taigu, Jinzhong 030801, China; cao13934643945@126.com

**Keywords:** weight estimation, deep learning, carbon footprint, precision agriculture, lightweight model

## Abstract

Addressing the carbon footprint in pig production is a fundamental technical basis for achieving carbon neutrality and peak carbon emissions. Only by systematically studying the carbon footprint can the goals of carbon neutrality and peak carbon emissions be effectively realized. This study aims to reduce the carbon footprint through optimized feeding strategies based on minimizing carbon emissions. To this end, this study conducted a full-lifecycle monitoring of the carbon footprint during pig growth from December 2024 to May 2025, optimizing feeding strategies using a real-time pig weight estimation model driven by deep learning to reduce resource consumption and the carbon footprint. We introduce EcoSegLite, a lightweight deep learning model designed for non-contact real-time pig weight estimation. By incorporating ShuffleNetV2, Linear Deformable Convolution (LDConv), and ACmix modules, it achieves high precision in resource-constrained environments with only 1.6 M parameters, attaining a 96.7% mAP50. Based on full-lifecycle weight monitoring of 63 pigs at the Pianguan farm from December 2024 to May 2025, the EcoSegLite model was integrated with a life cycle assessment (LCA) framework to optimize feeding management. This approach achieved a 7.8% reduction in feed intake, an 11.9% reduction in manure output, and a 5.1% reduction in carbon footprint. The resulting growth curves further validated the effectiveness of the optimized feeding strategy, while the reduction in feed and manure also potentially reduced water consumption and nitrogen runoff. This study offers a data-driven solution that enhances resource efficiency and reduces environmental impact, paving new pathways for precision agriculture and sustainable livestock production.

## Simple Summary

Pig farming is essential for the food supply, but it also contributes to greenhouse gas emissions. Manual weighing is labor-intensive and can cause stress to animals, potentially spreading disease. We developed a camera-based, non-contact system that utilizes a lightweight deep learning model (EcoSegLite) to segment the pig’s back in images and a machine learning model to estimate body weight in real-time. Using full-cycle monitoring of 63 pigs on a commercial farm, we integrated weight estimates with feeding adjustments and a life-cycle assessment of emissions. The system achieved accurate weight prediction (average error 3.2 kg) and enabled precision feeding that reduced feed use by 7.8%, manure output by 11.9%, and the carbon footprint per kilogram of live pig by 5.1%. Because the model is computationally light, it is suitable for deployment on farms and reduces handling, which benefits animal welfare. This approach offers a practical path to improve efficiency and lower emissions in sustainable pig production.

## 1. Introduction

The escalating levels of carbon emissions have precipitated severe environmental degradation, including global warming, ecosystem disruption, and increased climate variability, necessitating urgent emission reduction measures and making them a primary focus within the agricultural sector. This study aims to develop a contactless body weight estimation technology to reduce the carbon footprint of pork production and optimize feeding management based on minimizing carbon emissions, thereby enhancing industry sustainability. As a critical industry meeting human protein demands, pork production’s substantial carbon footprint has garnered widespread attention [1]. Agricultural activities account for approximately 14.5% of global greenhouse gas emissions [2], with pig farming significantly contributing through feed production, intestinal fermentation, and manure management. Achieving carbon neutrality and peak carbon emissions underscores the importance of monitoring carbon footprints and promotes the development of low-carbon pig farming technologies. These innovations leverage digital tools to enable precise resource management and process optimization, thereby alleviating environmental burdens [3]. However, theoretical prerequisites from the perspective of improving livestock production highlight the need for efficient feed conversion ratios (FCR) and optimized growth trajectories, which are critical for enhancing weight gain, reducing production costs, and minimizing disease risks [4]. Traditional livestock management relies heavily on labor-intensive weighing techniques, such as direct weighing, which are inefficient, costly, and pose risks of disease transmission among animals [5]. Such methods often result in suboptimal feed utilization, increasing feed waste and carbon emissions. Consequently, image-based contactless body weight estimation has emerged as a viable alternative, reducing manual intervention and the risk of disease transmission [6].

In recent years, benefiting from the latest advances in deep learning, computer vision technology has become an important driving force for revolutionizing livestock management, providing innovative approaches for achieving non-invasive monitoring and optimization [7,8]. In the field of non-contact body weight estimation, although research has made remarkable progress, numerous challenges and trade-offs remain in practical applications. To improve model accuracy, researchers have developed improved Mask R-CNN models that combine instance segmentation with regression techniques, achieving an Mean Average Precision (mAP) of 92% on diverse farm images, suitable for pig image processing in complex backgrounds [9]. Research combining multimodal technologies has also made progress, including the use of 3D point cloud reconstruction mesh models for cattle body weight estimation with a mean absolute error (MAE) of 2.5 kg [10]. In addition, the use of cross-attention visual Transformers with RGB-D images has enhanced the robustness of weight estimation, achieving an 88% correlation on pig datasets [11]; walk-through scale technology has achieved an MAE of 1.8 kg for finishing pigs in motion through dynamic weighing [12]; and body weight estimation methods based on depth cameras have reached MAEs as low as 2.0 kg in controlled environments, demonstrating the potential of multimodal data [13]. Furthermore, to address the complexity of imaging, one study proposed a cascaded vision-based weight estimation algorithm that significantly improved estimation accuracy [14]. To meet the need for deployment flexibility in dynamic farming environments, related research has also explored the rapid assessment of body weight in pigs using handheld mobile RGB-D cameras [15]. Although these methods perform excellently in terms of accuracy, their reliance on expensive hardware and complex data processing severely limits their large-scale deployment in commercial farms, and this practical challenge has prompted researchers to shift their focus toward lightweight solutions that can operate efficiently on edge devices.

To address the challenges of deployment efficiency, research has shifted toward lightweight models. For example, EfficientVit-C significantly reduces computational load by streamlining network structures, achieving a 90% correlation with actual body weight in controlled environments [16]. At the same time, the YOLO series has attracted considerable attention for its robust object detection capabilities in complex farming environments, with YOLOv8 achieving an mAP as high as 95.5% [17]. However, these lightweight models generally face challenges in accuracy and generalization capability. Studies have indicated that due to differences in farm environments, pig postures, and breeds, the generalization performance of models can decline significantly [18]. Furthermore, the computational load of most models still exceeds the thresholds required for edge deployment, particularly in terms of parameters and giga floating-point operations per second (GFLOPs), thereby impacting the feasibility of real-time applications. Meanwhile, beyond the optimization of weight estimation technologies themselves, the industry’s emission reduction goals also depend on the quantification of carbon footprints, and related research has thus gradually expanded to carbon footprint analysis and life cycle assessment.

In the field of carbon footprint research, some studies have focused on the macro level, such as analyzing the spatial and temporal characteristics and influencing factors of carbon emission efficiency in China’s pig industry to guide regional policy-making [19]. Other studies have approached the issue from a more specific perspective, using advanced tools such as Building Information Modeling (BIM) to assess the carbon and water footprints of pig house buildings [20]. There are also studies devoted to developing comprehensive methodologies for assessing the global warming potential (GWP) of pig production systems, quantifying their environmental impact by analyzing carbon and nitrogen balances [21]. However, a critical limitation across these studies is the lack of attention to the impact of excessive or irrational feeding, which has been estimated to increase carbon emissions by 8–20% due to intensified feed production and manure decomposition, as inferred from modeling studies of traditional pig farming practices where over-reliance on supplementary feed elevated emissions [22]. This oversight highlights the need for dynamic, real-time monitoring to address such inefficiencies. Most of these studies have focused on static, offline assessment and analysis, lacking effective integration with dynamic, real-time monitoring technologies, and therefore cannot provide immediate data support for daily production activities such as feed management, which limits their role in promoting actual emission reductions. This disconnection between monitoring and assessment makes it difficult for existing technologies to form a closed-loop system capable of guiding production in real time and quantifying the effects of emission reduction.

In summary, the current research presents a significant and unresolved gap: first, there is a lack of pig body weight estimation solutions that combine high-precision segmentation capability with lightweight characteristics; second, carbon footprint analysis largely remains at the level of static assessment and has yet to be integrated into a closed loop with real-time monitoring and feeding management. To fill this gap, it is necessary to develop an integrated, lightweight, and high-precision end-to-end solution that closely links real-time body weight monitoring with carbon footprint quantification, thereby achieving the dual goals of precision feeding and sustainable production.

To this end, this study proposes a lightweight instance segmentation model, EcoSegLite, and integrates it with a random forest weight estimation model to construct a “segmentation–weight estimation–feeding–carbon quantification” closed-loop system for precision feeding management and carbon reduction optimization. This technical system was validated in a case study at Pianguan Farm and demonstrated its practical effectiveness in improving sustainability. The main contributions of this work are as follows: the EcoSegLite model achieves extremely low computational cost (1.60 M parameters and 4.0 GFLOPs) while ensuring high accuracy (mAP50 = 96.7%, mAP50-95 = 82.3%); the weight estimation MAE reached 3.2 kg ($R^{2}$ = 0.92); more importantly, the precision feeding strategy based on this model resulted in a 7.8% reduction in feed consumption, an 11.9% reduction in manure output, and a 5.1% reduction in carbon footprint. The remainder of this paper is organized as follows: Section 2 presents the methods, Section 3 presents the results and analysis, Section 4 discusses the implications, and Section 5 concludes the paper and outlines future directions.

## 2. Materials and Methods

### 2.1. Dataset

The weight estimation dataset comprised 6420 images collected from Pianguan Farm (111°21′–112°00′ E, 39°12′–39°39′ N), Shanxi, between December 2024 and May 2025, covering the entire production cycle from piglets to market weight. These images were captured from multiple angles using a Hikvision DS-2CD3345D-I camera (Hangzhou Hikvision Digital Technology Co., Ltd., Hangzhou, China), with a resolution of 2560 × 1440 pixels, a frame rate of 25 frames per second, and stored in .MP4 format. The dataset comprised three pig breeds (Landrace, Jinfen White, and Duroc), totaling 63 pigs, with a weight range of approximately 6–121.7 kg, reflecting growth stages from weaned piglets to market-ready pigs. During the experiment, continuous monitoring was employed to periodically collect images of the pigs’ backs, with image capture scheduled every 3 days during the piglet stage (due to rapid growth rates) and every 7 days during the finishing stage (to balance accuracy and efficiency), validated through manual weighing benchmarks. In contrast, the traditional weighing system employed at the farm utilized manual platform scales with an accuracy of ±0.5 kg, requiring individual pig handling, weighing, and recording by farm staff. This process, averaging 11.2 s per pig, posed risks of stress and disease transmission due to physical contact, and was limited by labor-intensive operations and inconsistent environmental conditions. After data cleaning, images with complete body structures and appropriate lighting were retained. To support the carbon footprint assessment, full-lifecycle data on feed consumption, energy use, and manure management were collected from the same farm over a one-year production cycle. Feed consumption was recorded by an automatic feeding system equipped with weighing sensors, and net intake was verified with manual records from farm staff to exclude uneaten feed, reflecting changes in nutritional needs throughout the cycle. The feeding ration was formulated with an average caloric content of 12 MJ/kg, comprising approximately 70% corn, 20% soybean meal, and 10% other additives, adjusted weekly based on real-time weight estimates to optimize nutrient supply. Manure output was determined by collecting and weighing manure from the pig pens weekly using an electronic scale. The full-lifecycle weight estimation was integrated with feed consumption and manure output data to optimize feeding strategies and quantify the carbon footprint through a Life Cycle Assessment (LCA) framework.

### 2.2. Real-Time Weight Estimation Model

#### 2.2.1. Segmentation of Pig Back Images

Accurate segmentation of pigs from images is a critical prerequisite for precise weight estimation. To achieve this, a rigorous approach was adopted for data preparation and model application. A comprehensive dataset of pig back images was meticulously annotated using the Labelme tool, serving as the foundation for supervised model training. To evaluate model performance, the dataset was subjected to randomization to minimize sequential biases and enhance generalization, and then partitioned into training, validation, and test sets in an 8:1:1 ratio, comprising 5136 images for training and 642 images each for validation and testing. Before input into the segmentation model, all input images underwent standardized preprocessing. Preprocessing steps involved normalizing pixel intensity values and resizing images to consistent dimensions. Standardization was critical to ensuring image consistency across the dataset, enhancing input stability and accelerating convergence during training.

**Figure 1:**
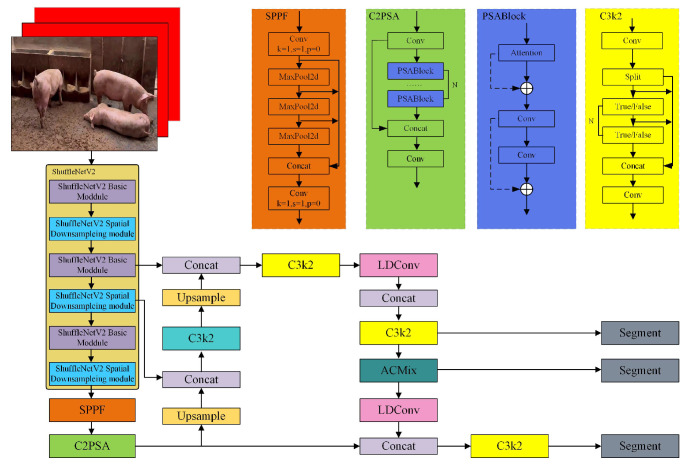
Architecture of EcoSegLite.

To achieve accurate segmentation of pig back images, a lightweight segmentation model, EcoSegLite, was proposed based on the characteristics of the dataset and the YOLO11-seg network architecture. The model architecture is shown in Figure 1, with specific improvements as follows:

(1) ShuffleNetV2 was employed as the backbone network, leveraging its channel shuffling mechanism to enhance feature representation while reducing computational complexity through group convolutions, optimizing memory usage efficiency, and making it suitable for resource-constrained environments.
(2) WConvolutions in the neck were replaced with Linear Deformable Convolution (LDConv), which dynamically adjusts the sampling shape of convolution kernels to improve the flexibility and accuracy of feature extraction while reducing model parameters and computational overhead.
(3) ACmix was introduced as an attention mechanism, combining the global perception of self-attention with the local feature extraction capabilities of convolutions, reducing redundant computations through shared processing to enhance model performance at low computational cost.

The EcoSegLite model was trained on a computer with the following hardware configuration: Windows 11 Professional operating system, 12th Gen Intel(R) Core(TM) i5-12400F processor (base frequency 2.50 GHz), and NVIDIA GeForce RTX 2070Super GPU. The experiment was programmed using Python 3.9.2, with the model developed based on the PyTorch 2.0.1 framework. To enhance training speed, CUDA 11.7 was employed. Input training images were resized to 640 × 640 × 3 to balance computational efficiency and feature resolution, a setting widely adopted in real-time object detection tasks. Based on empirical testing, a batch size of 8 was selected to ensure GPU memory usage stability while maintaining training convergence speed. Training utilized the Adaptive Moment Estimation (Adam) optimizer, which adaptively adjusts the learning rate for each parameter by estimating first- and second-order moments of gradients, combining the advantages of momentum and RMSProp. This optimizer is particularly suitable for moderately sized datasets with moderate variability, providing faster convergence and better generalization. The initial learning rate was set to 0.01, determined through preliminary experiments to achieve rapid convergence in early training stages without inducing instability. A step decay strategy was adopted, reducing the learning rate by a factor of 10 every 50 epochs to fine-tune model weights in later stages and prevent overfitting. Training was conducted for a total of 200 epochs, sufficient for convergence, as evidenced by stable mAP curves and low validation loss in the final epochs. The best model checkpoint was selected based on validation set performance.

The EcoSegLite model processes input images through the following steps: First, the ShuffleNetV2 backbone extracts multi-scale features from input images, providing rich representations for pig back regions. Next, the LDConv module adjusts the sampling positions of convolution kernels, focusing on relevant features to enhance the model’s ability to handle morphological variations. Then, the ACmix module integrates global and local features to generate refined feature maps, emphasizing pig back regions. Finally, the decoded feature maps produce the final segmentation mask, accurately distinguishing pig back regions from the background. Compared to traditional segmentation methods, EcoSegLite achieves higher accuracy, particularly under complex farm conditions. Its lightweight design, enabled by ShuffleNetV2, reduces computational demands, making it deployable on edge devices commonly used in agricultural settings. Additionally, the model’s adaptability to morphological variations ensures consistent performance across different pig breeds and growth stages. The segmented pig back images serve as input for the weight estimation algorithm, extracting key features such as body area and contours. By providing precise segmentation masks, EcoSegLite enhances the accuracy of subsequent weight predictions, contributing to more effective farm management and resource optimization. The EcoSegLite model offers a robust and efficient solution for pig back image segmentation, leveraging advanced deep learning techniques to overcome challenges in real farm environments. Its integration into the weight estimation pipeline marks a significant advancement in precision livestock farming.

#### 2.2.2. Feature Extraction

After completing the segmentation of pig back images, morphological closing operations and hole filling were applied to the binary masks to ensure connected foreground regions and eliminate small noise artifacts. Based on the refined masks, five key features were defined and calculated from a geometric perspective [23] to thoroughly characterize the relationship between back contours and weight, providing effective support for weight estimation.

Relative Projection Area of Pig’s Back (RA): RA is the ratio of the pig’s back area in the mask image to the total area of the binary image, calculated as follows:

(1) $$RA=\frac{P_{w}}{P_{t}}$$

Pig Back Contour Perimeter (CP): CP is the total length of the pig’s back boundary in the mask image, representing the total number of pixels along the pig’s body contour. This parameter quantifies the contour extent and provides insights into shape complexity. The calculation is as follows:

(2) CP=cv2.arcLengthcontours,True

where contours are obtained using cv2.findContours, and ‘True’ indicates that the contour is closed [24].

Body Length (BL) and Body Width (BW): BL is the length of the longest side of the minimum bounding rectangle enclosing the pig’s back in the mask image, derived from the longest axis of the minimum enclosing rectangle. This parameter is a key indicator of the pig’s total length. BW is the maximum lateral dimension of the pig’s body, corresponding to the short axis of the minimum enclosing rectangle. This parameter provides insights into the pig’s width or girth. The calculations are as follows:

(3) $$BL=\sqrt{{\left(P_{2x}-P_{1x}\right)}^{2}+{\left(P_{2y}-P_{1y}\right)}^{2}}$$

(4) $$BW=\sqrt{{\left(P_{1x}-P_{0x}\right)}^{2}+{\left(P_{1y}-P_{0y}\right)}^{2}}$$

where *P* represents the coordinates of the four corners of the minimum bounding rectangle returned by cv2.minAreaRect(contours). The Euclidean distance formula is used to precisely calculate the pig’s body length and width.

Ellipse Eccentricity (E): E is calculated by extracting the pig’s back contour from the mask image, fitting an ellipse using the least squares method to describe the contour, and computing the eccentricity as the square of the difference between the major and minor axes of the ellipse. The calculation is as follows:

(5) $$E=\sqrt{1-{\left(\frac{b}{a}\right)}^{2}}$$

where *a* is the major axis of the ellipse, and *b* is the minor axis.

#### 2.2.3. Pig Weight Estimation

After successfully segmenting pig back regions and extracting key morphological features using the EcoSegLite model, this study developed a machine learning model to achieve non-contact, high-precision pig weight estimation. Considering the low dimensionality of input features (5 dimensions) and the potentially complex nonlinear relationships between weight and morphological features, the Random Forest Regressor (RFR) algorithm was selected as the core estimation model.

Random forests, as an ensemble learning algorithm, integrate the results of multiple decision trees, typically offering higher prediction accuracy and better generalization than single decision trees while effectively avoiding overfitting [25]. This model excels in handling low-dimensional, numerical feature data, making it suitable for the 5-dimensional feature parameters extracted from mask images in this study. The relationship between pig physiological morphology and weight is not simply linear, and random forests can effectively capture such complex nonlinear dependencies [26]. Random forests use the Bootstrap sampling method to draw multiple subsamples with replacement from the original dataset, constructing a decision tree for each subsample. During node splitting in decision tree construction, the model randomly selects a subset of features for optimal splitting. For regression tasks, the final prediction is the average of all decision tree predictions.

The model’s input is a 5-dimensional feature vector (X), extracted from the segmentation mask of each pig back image as described in Section 2.2.2. It is defined as follows:

(6) $$X=[f_{1},f_{2},f_{3},f_{4},f_{5}]$$

where $f_{1}$ to $f_{5}$ represent the five selected key feature parameters.

The model’s output is a scalar *Y*, representing the estimated pig weight (in kg). This estimated value is compared with the true weight measured by electronic scales to evaluate model performance.

To train the model and conduct an unbiased evaluation, and to prevent information leakage that could occur if images of the same pig appeared in both the training and test sets, the dataset was partitioned by individual pig ID. Images from different individuals were strictly assigned to the training, validation, and test sets without overlap. The split ratio was 80% for training and 20% for testing. The training set was used for parameter learning, while the test set was used to evaluate the final generalization performance of the model. To achieve optimal model performance, key hyperparameters of the random forest model were optimized using 5-fold cross-validation combined with grid search. The optimized hyperparameters included the number of decision trees (n_estimators), maximum tree depth (max_depth), and minimum samples required for node splitting (min_samples_split).

### 2.3. Feeding Management Optimization Strategy Based on Weight Estimation

This study integrated real-time body weight estimation technology into the optimization of feeding management strategies to achieve efficient resource utilization and reduce environmental impacts. The technology framework, centered on the EcoSegLite model, provided precise individual body weight data to enable dynamic adjustment of feeding schemes. At Pianguan Pig Farm, 63 pigs were monitored and randomly assigned to an experimental group (*n* = 43) and a control group (*n* = 20), stratified by sex, initial body weight, and breed proportion. The experimental group was managed using the precision feeding strategy developed in this study, leveraging automated non-contact weight estimation via the EcoSegLite model, while the control group was managed using the farm’s conventional feeding regimen, which involved daily manual weighing and fixed feed allocation based on average weight estimates. Both groups were maintained under identical housing conditions (pen area, stocking density, temperature, humidity, and ventilation) and management practices, differing only in the feeding strategy applied. Under the guidance of livestock nutrition experts, feeding frequency and diet composition were adjusted according to the nutritional requirements of pigs at different growth stages, providing a theoretical basis for optimization. The manual weighing process for the control group required approximately 2 h per day (averaging 11.2 s per pig), totaling 300 h over 150 days, whereas the experimental group’s automated approach reduced this to 15 min per day (0.14 s per pig), totaling 37.5 h, representing a labor time saving of approximately 87.5% (262.5 h). Specific details are provided in Table 1.

Table 1 presents the feeding frequencies and feed compositions for pigs at different growth stages, providing a practical basis for the optimization strategies employed in this study. Building upon this foundation, the EcoSegLite model further optimized feed ratios through real-time weight estimation. For instance, during the rapid growth phase (30–80 kg), the model adjusted the corn ratio from 63% to 68% and the soybean meal ratio from 16% to 18–20% based on precisely monitored weight data; for pigs with slower growth (daily weight gain < 0.6 kg), the model identified these individuals and increased the soybean meal ratio to 20% with an additional 2% fishmeal, in accordance with the Chinese Feeding Standards (2020 edition), to ensure the fulfillment of energy and protein requirements.

Utilizing the EcoSegLite model and random forest regression methods, the research team leveraged full-cycle weight monitoring data from 63 pigs at Pianguan Farm between December 2024 and May 2025, integrated with feed consumption and manure output data, to support dynamic feeding optimization from piglets to market weight. The high-precision weight data from the EcoSegLite model proved critical to this process, and when combined with the expert-recommended feed intake plan, it facilitated the construction of growth curves that validated the enhancement of growth performance through optimized feeding. This approach transcended the limitations of traditional group feeding by relying on the model’s real-time monitoring of individual morphological features—such as relative area (RA), chest circumference (CP), body length (BL), body width (BW), and ellipse eccentricity (E)—to provide accurate analyses of individual metabolic demands. Researchers accordingly adjusted feed components and feeding amounts in a timely manner; for example, increasing protein proportions when weight gain slowed and reducing feed quantities when weight gain accelerated, ensuring precise alignment between nutritional supply and physiological needs.

The integration of expert recommendations with the EcoSegLite model’s real-time weight estimation significantly enhanced the precision and efficiency of feeding management. By precisely matching feed supply to individual physiological demands, this study improved resource utilization efficiency and supported environmental sustainability. The reduction in feed consumption directly lowered carbon emissions associated with feed production, while the decrease in manure output further mitigated the environmental impact of manure management. Moreover, these environmental benefits were achieved without compromising the normal growth of the pigs, as veterinary assessments and production indicators confirmed that the physiological status and weight gain of the pigs remained within normal ranges. This precision feeding model fully demonstrated the potential of combining the EcoSegLite model with expert knowledge, providing a scalable paradigm for sustainable pig farming.

### 2.4. Carbon Footprint Calculation Method for Pig Farming

To comprehensively quantify the environmental impact of pig farming and evaluate the contribution of real-time weight estimation to resource optimization and sustainability, this study adopted a Life Cycle Assessment (LCA) method. This involved analyzing the carbon footprint based on data from the annual production cycle at Pianguan Farm from December 2024 to May 2025.

#### 2.4.1. LCA Framework: Scope and Data Collection

The system boundary for this assessment was defined as “cradle-to-farm-gate”, encompassing feed production (cultivation, processing, transportation), farm operations, energy use, pig digestion and metabolism, and manure management, with the functional unit set as the carbon footprint per kilogram of pig (kg ${\mathrm{CO}}_{2}$-eq/kg). This scope focused on quantifying greenhouse gas emissions contributing to the carbon footprint.

The carbon footprint data for this study were entirely derived from our one-year production cycle monitoring at a farm in Pianguan, Shanxi. The subjects monitored included Landrace, Jinfen White, and Duroc pigs, ensuring the comprehensiveness and representativeness of the data. Data collection adhered strictly to the 2019 Intergovernmental Panel on Climate Change (IPCC) guidelines and the Chinese Life Cycle Database (CLCD) standards [27], aiming to establish a precise and reliable foundation for carbon footprint assessment. The collected data covered key emission sources throughout the pigs’ production cycle. We used sensors and on-site records to precisely monitor feed consumption. The feed formula consisted of corn (63%), soybean meal (16%), wheat bran (12%), and other components (9%), with detailed consumption amounts provided in Table 2. Energy consumption data, including electricity for lighting, ventilation, and heating (0.88 t ${\mathrm{CO}}_{2}$-eq/MWh), and fuel for transportation and equipment (3.18 t ${\mathrm{CO}}_{2}$-eq/t diesel), were also precisely collected through farm sensors and recording systems. Furthermore, we recorded manure management data, including yield, storage methods (solid/liquid), and treatment processes (anaerobic digestion, composting), with corresponding emission factors detailed in Table 3 and Table 4. By integrating on-site surveys and sensor monitoring, this study ensured the accuracy and reliability of all data, providing a solid basis for subsequent carbon footprint evaluations.

#### 2.4.2. Carbon Footprint Calculation Methodology

The carbon footprint quantifies greenhouse gas emissions (${\mathrm{CO}}_{2},{\mathrm{CH}}_{4},N_{2}O$) expressed in ${\mathrm{CO}}_{2}$ equivalents (kg ${\mathrm{CO}}_{2}$-eq), based on IPCC 2019 guidelines (Global Warming Potential: ${\mathrm{CO}}_{2}$ = 1, ${\mathrm{CH}}_{4}$ = 28, $N_{2}O$ = 298). The total carbon footprint (CFT) is the sum of emissions from feed production, transportation, enteric fermentation, energy use, and manure management:

(7) $$CFT=CF_{forage},CF_{transport},CF_{enteric},CF_{energy},CF_{manure}$$

The carbon footprint per unit live weight (CF) is calculated by normalizing the total carbon footprint to the total live weight of pigs produced annually ($W_{live}$):

(8) $$CF=\frac{CFT}{W_{live}}$$

Feed production and processing emissions ($CF_{forage}$) include corn (0.37 kg ${\mathrm{CO}}_{2}$-eq/kg), soybean meal (0.11 kg ${\mathrm{CO}}_{2}$-eq/kg), and wheat bran (0.26 kg ${\mathrm{CO}}_{2}$-eq/kg), representing the sum of emissions from crop cultivation and processing:

(9) $$CF_{firage}=\sum_{i}\left(CF_{c,i}+CF_{p,i}\right)$$

Cultivation emissions ($CF_{c}$) account for fertilizer production (nitrogen fertilizer 2.12 t ${\mathrm{CO}}_{2}$-eq/t, phosphate fertilizer 0.64 t ${\mathrm{CO}}_{2}$-eq/t, potash fertilizer 0.18 t ${\mathrm{CO}}_{2}$-eq/t), $N_{2}O$ emissions from nitrogen fertilizer application (direct emission factor 0.01 t $N_{2}O$-N/t N, indirect 0.003 t $N_{2}O$-N/t volatile N, volatilization rate 0.05 t volatile N/t applied N), urea decomposition (0.02 t ${\mathrm{CO}}_{2}$/t urea), pesticides (12.44 t ${\mathrm{CO}}_{2}$-eq/t), agricultural film (22.72 t ${\mathrm{CO}}_{2}$-eq/t), irrigation electricity (corn 368.7 kWh/ha, soybean 38.7 kWh/ha, wheat 839.4 kWh/ha), and mechanical fuel (corn 67.85 L/ha, soybean 45.96 L/ha, wheat 78.8 L/ha). Processing emissions ($CF_{p}$) are based on energy emission factors (electricity 0.88 t ${\mathrm{CO}}_{2}$-eq/MWh, diesel 3.18 t ${\mathrm{CO}}_{2}$-eq/t), with by-product emissions allocated using the energy allocation method (soybean meal 13.82 MJ/kg, wheat bran 4.15 MJ/kg).

Transportation emissions ($CF_{transport}$) are calculated based on transportation distance, tonnage, and vehicle type emission factors:

(10) $$CF_{transport}=\left(L_{forage}\times T_{forage}+L_{pig}\times T_{pig}\right)\times EF_{transport}$$

where $L_{forage}$ is the feed transportation distance, $T_{forage}$ is the feed transportation volume, $L_{pig}$ is the live pig transportation distance, $T_{pig}$ is the live pig transportation volume, and $EF_{transport}$ is the transportation emission factor (light vehicles 0.185 kg ${\mathrm{CO}}_{2}$-eq/km, medium vehicles 0.197 kg ${\mathrm{CO}}_{2}$-eq/km, heavy vehicles 0.321 kg ${\mathrm{CO}}_{2}$-eq/km).

Enteric fermentation emissions ($CF_{enteric}$) are calculated based on the number of pigs ($N_{m}$), methane emission factor ($EF_{enteric,CH_{4},m}$), and the GWP of ${\mathrm{CH}}_{4}$:

(11) $$CF_{enteric}=\sum_{m}\left(EF_{enteric,CH_{4},m}\times N_{m}\times GWP_{CH_{4}}\right)\times Days/365$$

where $N_{m}$ is the number of pigs, and $GWP_{CH_{4}}$ is the global warming potential of methane. The methane emission factor is adjusted based on the average weight of each pig breed (Landrace 75 kg, Jinfen White 75 kg, Duroc 76 kg) relative to baseline factor $EF_{enteric,CH_{4}}$:

(12) $$EF_{enteric,CH_{4},m}=EF_{enteric,CH_{4}}\times W_{m}/105$$

where $W_{m}$ represents the average body weight of pigs. A value of 105 kg is used as the average body weight of market pigs. This is because the enteric methane emission coefficients vary among different pig breeds and are positively correlated with body weight and growth rate. Given the difficulty in accurately quantifying growth rate, the coefficient is determined based on the ratio of the average weight of different pig breeds to the average weight of market pigs. The baseline enteric methane emission factor ($EF_{enteric,CH_{4}}$) for market pigs is established at 1.00 kg/head/year, serving as the reference factor [28].

Energy use emissions ($CF_{energy}$) are derived from electricity, coal, diesel, and their respective emission factors:

(13) $$CF_{energy}=\sum_{j}\left(E_{energy,j}+Q_{energy,j}\right)$$

where $E_{energy,j}$ represents the consumption of energy source j, and $Q_{energy,j}$ is the corresponding emission factor; specifically, electricity is 0.88 t ${\mathrm{CO}}_{2}$-eq/MWh, coal is 2.05 t ${\mathrm{CO}}_{2}$-eq/t, and diesel is 3.18 t ${\mathrm{CO}}_{2}$-eq/t.

Manure management emissions ($CF_{manure}$) include ${\mathrm{CH}}_{4}$ and $N_{2}O$, considering their respective emission factors and Global Warming Potentials (GWPs) (Table 3: ${\mathrm{CH}}_{4}$ emission factor of 0–0.005 kg ${\mathrm{CH}}_{4}$/kg N, $N_{2}O$ emission factor of 0.01–0.1 kg $N_{2}O$-N/kg N, volatilization rate of 0.01 t volatile N/t applied N, and leaching rate of 0.05 t leached N/t applied N).

(14) $$CF_{manure}=\sum_{m}\left(EF_{CH_{4},m}\times N_{m}\times GWP_{N_{2}O}+N_{2}O_{D}\times GWP_{N_{2}O}+N_{2}O_{IN}\times GWP_{N_{2}O}\right)$$

where $EF_{CH_{4},m}$ represents the methane emission factor from manure management, $N_{2}O_{D}$ represents the direct $N_{2}O$ emission, and $N_{2}O_{\mathrm{IN}}$ represents the indirect $N_{2}O$ emission.

## 3. Results

### 3.1. Segmentation Performance of Pig Back Images

**Figure 2:**
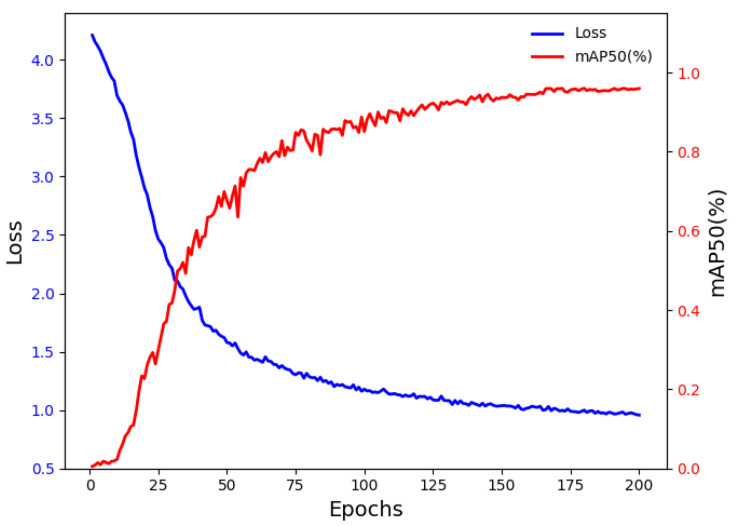
Training dynamics of EcoSegLite over epochs.

**Figure 3:**
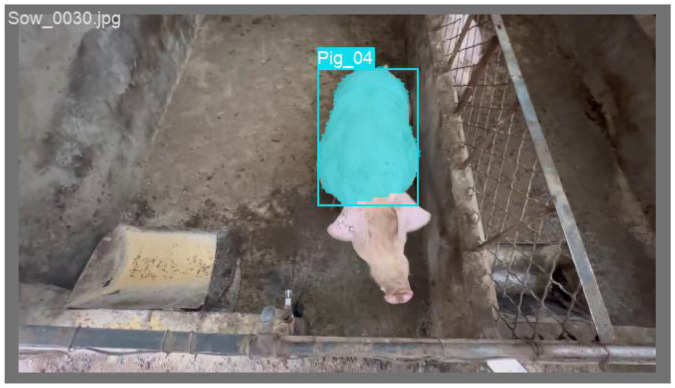
Example of pig segmentation visualization.

Figure 2 illustrates the training dynamics of the EcoSegLite model, depicting the changes in loss and mAP50 across epochs. Initially, the loss curve decreases rapidly, indicating effective learning in the early stages. As training progresses, the loss stabilizes, suggesting that the model has converged. Minor fluctuations near the end of training may reflect typical variations in the learning process. The mAP50 curve rises sharply during the early epochs, reflecting significant improvements in detection accuracy. It subsequently levels off at a high value, indicating consistent model performance in detecting pigs for weight estimation. These trends confirm that the model has converged by the final epochs, with both loss and mAP50 metrics demonstrating the effectiveness of the training process. Figure 3 shows the segmentation results of the pig’s back produced by the model.

#### 3.1.1. Ablation Study

An ablation study was conducted to evaluate the individual and combined impacts of the architectural components—ShuffleNetV2, LDConv, and ACmix—on the YOLO11 model (used as the baseline for pig back image segmentation). The baseline model had 2.59 M parameters, 6.4 GFLOPs of computational performance, and a frame rate of 99.4 FPS, serving as a reference for assessing changes in computational complexity and performance.

Incorporating ShuffleNetV2 reduced the parameter count to 1.71 M and GFLOPs to 4.1, improving computational efficiency but lowering FPS to 80.2, indicating a trade-off between reduced computational complexity and inference speed due to its lightweight operations’ integration with YOLO11. Adding LDConv alone reduced parameters to 2.17 M and GFLOPs to 5.5, with an FPS of 85.2, suggesting partial optimization of convolution operations, though interactions with the baseline architecture limited speed improvements. In contrast, integrating ACmix increased parameters to 2.60 M and GFLOPs to 6.5 but boosted FPS to 103.5, likely due to enhanced efficiency in feature extraction and spatial-channel processing.

Combining ShuffleNetV2, LDConv, and ACmix further reduced parameters to 1.60 M and GFLOPs to 4.0, achieving an FPS of 98.2, close to the baseline level. This indicates a synergistic effect on computational efficiency, though the slight FPS decrease highlights the complex interplay of components affecting inference speed. Detection accuracy (measured by mAP50 and mAP50-95) significantly improved under the full configuration, reaching 96.7% and 82.3%, respectively, showing clear improvements over the baseline, with ACmix contributing substantially to this enhancement. Detailed performance metrics for each configuration are presented in Table 5, supporting the observed trends.

#### 3.1.2. Comparison Results with Different Models

**Figure 4:**
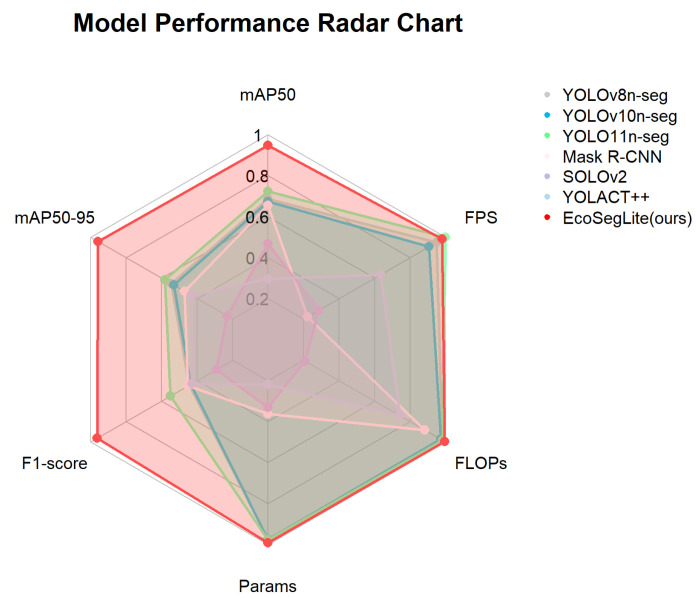
Comprehensive performance comparison of six segmentation algorithms.

To evaluate the performance of the EcoSegLite model in pig back image segmentation, we conducted performance assessments on multiple models, including YOLOv8 [29], YOLOv10 [30], YOLO11, Mask R-CNN [31], SOLOv2 [32], and YOLACT++ [33], using the same dataset as EcoSegLite. As shown in Table 6, compared to the YOLO11 model, EcoSegLite improved mAP, mAP50-95, and F1-score by 3.5%, 6.1%, and 5.4%, respectively, while reducing both parameter count and GFLOPs. A comprehensive performance comparison of different detection algorithms is shown in Figure 4, where data were normalized for visualization. The normalization method used maximum value normalization, where each metric was divided by its maximum value across all models, resulting in values ranging from 0 to 1 for intuitive comparison of relative performance. Table 6 summarizes the detailed experimental results.

EcoSegLite demonstrated superior accuracy across all evaluation metrics, surpassing YOLOv8n-seg (92.6% mAP50, 75.6% mAP50-95, 85.2% F1-score) and YOLOv10n-seg (92.4%, 75.4%, 85.1%) by 4.1–4.3% in mAP50. It also outperformed Mask R-CNN (92.1%, 74.4%, 85.3%) by 4.6% in mAP50. EcoSegLite’s lightweight design, achieved through the integration of ShuffleNetV2, LDConv, and ACmix, minimized computational demands while maintaining a competitive FPS of 98.2, nearly matching YOLO11n-seg’s 99.4. In contrast, SOLOv2 (89.2% mAP50, 46.25 M parameters, 52.3 FPS) and YOLACT++ (86.5% mAP50, 53.72 M parameters, 75.2 FPS) exhibited lower accuracy and slower inference speeds, reflecting their higher computational overhead.

The trade-off between accuracy and efficiency is evident: YOLOv8s-seg and YOLOv10s-seg (9.83 M and 8.19 M parameters, 82.8 and 84.6 FPS) offered moderate accuracy gains (93.2% and 93.1% mAP50) at the expense of speed, making them less suitable for real-time applications. Mask R-CNN and SOLOv2, with 44.12 M and 46.25 M parameters, respectively, achieved lower FPS (48.0 and 52.3), limiting their scalability. EcoSegLite’s balanced performance highlights its optimization for resource-constrained environments.

### 3.2. Accuracy of Weight Estimation

**Figure 5:**
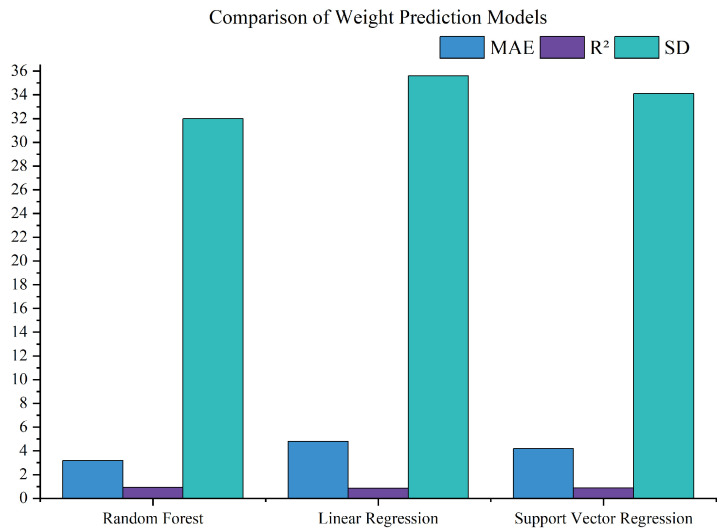
Comparison of weight prediction models.

To evaluate the impact of different models on weight prediction, a comparative analysis was conducted using random forest, linear regression, and support vector regression (SVR) on the same dataset, as shown in Figure 5. Random forest outperformed the alternatives, achieving an MAE of 3.2 kg and $R^{2}$ of 0.92, compared to linear regression (MAE 4.8 kg, $R^{2}$ 0.85) and SVR (MAE 4.2 kg, $R^{2}$ 0.88), demonstrating superior accuracy in capturing weight variability (SD 32.0 kg vs. 35.6 kg for linear regression and 34.1 kg for SVR). This enhanced prediction accuracy directly influenced subsequent steps by improving the reliability of real-time weight-based decision-making, enabling more effective resource allocation and supporting the optimization strategies that reduce the carbon footprint.

The combination of the EcoSegLite segmentation results with the random forest regression model achieved a mean absolute error (MAE) of 3.2 kg (95% CI: 2.8–3.6 kg) and a coefficient of determination ($R^{2}$) of 0.92 (*p* < 0.001) on the test set for the experimental group (*n* = 43), with individual weight estimates ranging from 6 kg to 121.7 kg, focusing on the growing-finishing phase (48.3–121.7 kg) due to slightly lower segmentation accuracy for piglets. The standard deviation of these estimates was 32.0 kg, reflecting variability influenced by the estimation process. In contrast, for the control group (*n* = 20) managed with traditional manual weighing, recorded weight data revealed a range of 8 kg to 118.5 kg, with a standard deviation of 28.0 kg.

Feature importance analysis revealed that relative projected area (RA) contributed the most (34.6%), followed by body length (BL, 22.4%), body width (BW, 19.8%), contour perimeter (CP, 14.7%), and elliptical eccentricity (E, 8.5%), consistent with their physical correlations with body weight. Table 7 summarizes detailed feature statistics from the Pianguan dataset, where RA varied from 10.2% to 49.8% (mean 28.4%, standard deviation 9.1%), supporting its role as a key predictive indicator. Contour perimeter (CP) ranged from 69.8 cm to 189.3 cm (mean 127.6 cm, standard deviation 28.4 cm), reflecting shape variability, while body length (BL) and body width (BW) averaged 98.7 cm and 53.9 cm, respectively (ranges 70.5–132.1 cm and 31.4–79.3 cm), aligning with the 50–121.7 kg weight range (correlation coefficients r = 0.55 and r = 0.50). Elliptical eccentricity (E) averaged 0.49 (range 0.21–0.79), exhibiting the lowest correlation with weight (r = 0.35), indicating a limited direct contribution but offering supplementary value in characterizing shape differences.

To assess the model’s generalization ability, cross-validation was performed using datasets from Fengyang Farm and Puxian Farm, collected from February to May 2025, covering diverse breeds and management practices. The Fengyang dataset included 48 pigs (primarily Landrace and Yorkshire, weight range 50.2–118.4 kg) in semi-open pig houses with light intensity of 500–800 lux. The Puxian dataset included 42 pigs (primarily Landrace and Yorkshire, weight range 47.8–123.5 kg) in enclosed pig houses with light intensity of 300–600 lux and high posture variability (approximately 30% of images showed lying or partially occluded pigs). The model achieved an MAE of 3.5 kg, $R^{2}$ of 0.91, mean absolute percentage error (MAPE) of 2.6%, and root mean square error (RMSE) of 4.3 kg at Fengyang Farm; at Puxian Farm, it achieved an MAE of 3.7 kg, $R^{2}$ of 0.89, MAPE of 2.8%, and RMSE of 4.6 kg (Table 8). The higher MAE and MAPE at Puxian Farm were attributed to posture variability, with approximately 30% of images affected by lying or occlusion, leading to a segmentation accuracy decline (mAP50 reduced by approximately 5.2%, consistent with Section 4.1 analysis), suggesting the need for posture augmentation or multimodal data to enhance robustness in complex scenarios. These results indicate strong generalization ability, though improvements for specific farm environments are necessary.

Hyperparameter optimization for the random forest model was conducted using the Pianguan Farm dataset (6420 images, 63 pigs) with 5-fold cross-validation and grid search, testing parameters including the number of decision trees (n_estimators = 50, 100, 200, 500), maximum depth (max_depth = 10, 20, 30, None), and minimum samples for splitting (min_samples_split = 2, 5, 10). The optimal configuration was n_estimators = 200, max_depth = 20, min_samples_split = 5, achieving the lowest validation MAE (3.2 kg) and highest R^2^ (0.92). Compared to other configurations, this setup effectively balanced model complexity and generalization, avoiding overfitting. To ensure applicability at Fengyang and Puxian Farms, the optimal configuration was further validated in cross-validation, with MAE and $R^{2}$ fluctuations of ±0.3 kg and ±0.02, respectively, indicating model stability across datasets. These optimization results support the model’s high-precision predictions at Pianguan Farm and cross-validation datasets, providing a reliable technical foundation for precision feeding.

### 3.3. Impact of Feeding Optimization on Carbon Emissions

Based on the full-cycle body weight estimation results obtained from the EcoSegLite segmentation and random forest regression models, a feeding optimization strategy with dynamic adjustments to feed quantity and composition was implemented in the experimental group to improve feed utilization efficiency and reduce waste. Compared with the control group, the optimized feeding reduced the total feed consumption by an average of 7.8% (*p* < 0.05), decreased daily manure output by 11.9% (*p* < 0.05), and significantly lowered carbon emissions. The full-cycle monitoring data ensured the precision of feeding adjustments and provided a reliable basis for the quantitative assessment of the carbon footprint.

**Figure 6:**
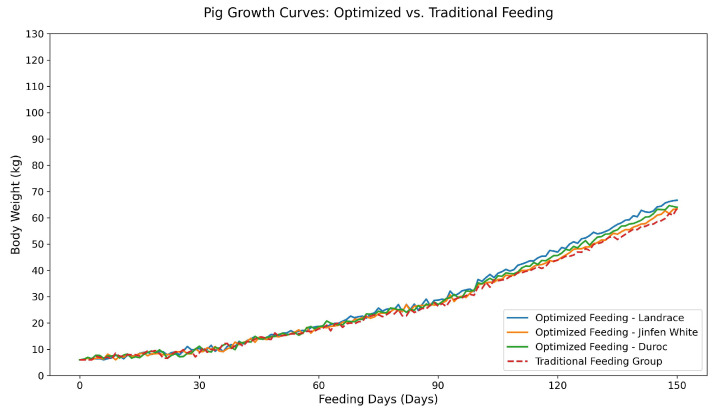
Growth curves of experimental and control pigs based on full-cycle body weight monitoring (*n* = 63, December 2024–May 2025).

To further validate the impact of feeding optimization on pig growth, this study constructed growth curves for both the optimized and conventional feeding groups based on full-lifecycle weight monitoring data (Figure 6). The results indicate that the average growth rate of the optimized feeding group (average daily gain of approximately 0.65 kg/day) was comparable to or slightly higher than that of the conventional group (0.63 kg/day), demonstrating that optimized feeding maintained growth performance while reducing feed consumption by 7.8% (*p* > 0.05, *t*-test). Figure 6 illustrates the average body weight curves over time for the optimized and control groups, confirming that the precision feeding strategy based on full-lifecycle weight monitoring effectively supported sustainable production.

Quantitative analysis based on the Life Cycle Assessment framework revealed a corresponding reduction in carbon emissions. Feed production emissions, the primary contributor accounting for approximately 50% of the baseline total, decreased by 6.5% due to reduced feed demand. Enteric fermentation emissions, with a baseline rate of 1.5 kg methane per head annually, decreased by 4.2% due to improved growth efficiency and shortened fattening periods. Manure management emissions decreased by 5.3% due to reduced manure volumes. Based on an average sample weight of 74.9 kg, these changes collectively reduced the overall carbon footprint per kilogram of live pig production by 5.1%.

Compared to the control group using traditional feeding methods, which had an average emission of 8.3 kg ${\mathrm{CO}}_{2}$-eq/kg pig, the optimized approach reduced emissions to 7.9 kg ${\mathrm{CO}}_{2}$-eq/kg pig. This improvement was statistically significant (*p* < 0.05, paired *t*-test, *n* = 63). The emission reduction was most pronounced during the fattening phase, as precision feeding effectively minimized overfeeding. However, these results are specific to the studied breeds (Landrace, Jinfen White, and Duroc) and the conditions at Pianguan Farm, suggesting the need for further validation in different environments to assess broader applicability.

**Figure 7:**
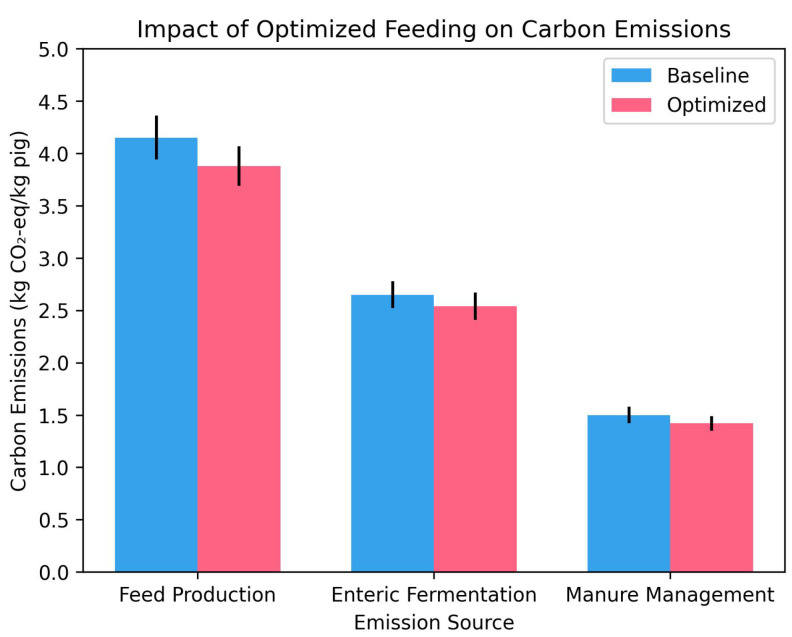
Carbon emissions comparison between baseline and optimized feeding schemes.

Figure 7 illustrates the carbon emissions of baseline and optimized feeding schemes across three categories: feed production, enteric fermentation, and manure management (unit: kg ${\mathrm{CO}}_{2}$-eq/kg pig). Baseline emissions were 4.15 kg ${\mathrm{CO}}_{2}$-eq/kg pig for feed production, 2.65 kg ${\mathrm{CO}}_{2}$-eq/kg pig for enteric fermentation, and 1.50 kg ${\mathrm{CO}}_{2}$-eq/kg pig for manure management, totaling 8.3 kg ${\mathrm{CO}}_{2}$-eq/kg pig. Optimized feeding reduced these to 3.88 kg ${\mathrm{CO}}_{2}$-eq/kg pig, 2.54 kg ${\mathrm{CO}}_{2}$-eq/kg pig, and 1.42 kg ${\mathrm{CO}}_{2}$-eq/kg pig, respectively, totaling 7.9 kg ${\mathrm{CO}}_{2}$-eq/kg pig. Error bars represent the standard error of the mean (SEM) based on carbon footprint assessments of 63 pigs from December 2024 to May 2025 (each pig assessed using feed consumption, energy use, and manure management data over the entire production cycle, calculated via the LCA framework). The SEM is calculated as $SEM=\frac{SD}{\sqrt{n}}$, where SD is the standard deviation of the carbon footprint data, and n is the number of pigs (*n* = 63).

## 4. Discussion

### 4.1. Evaluation of Segmentation and Weight Estimation Techniques

The segmentation and weight estimation performance presented in the Section 3 reflected the effectiveness of the EcoSegLite model in recognizing and extracting features from dorsal images of pigs. As shown in Table 7, the model accurately delineated the dorsal region across different growth stages, with the relative projection area (RA) ranging from 10.2% to 49.8%, demonstrating stable adaptability to variations in body conformation. By integrating the ShuffleNetV2, LDConv, and ACmix modules into the network architecture, the efficiency and adaptability of feature extraction were significantly enhanced. This outcome was consistent with findings from studies on lightweight convolutional neural networks for resource-constrained environments in agricultural computer vision [34], while achieving higher segmentation accuracy at a lower computational complexity in the present study.

The high-precision segmentation provided critical morphological features for subsequent body weight estimation. In this study, the random forest regression model, based on the segmentation results, achieved a mean absolute error (MAE) of 3.2 kg and an $R^{2}$ of 0.92 across a weight range of 48.3–121.7 kg, indicating strong predictive capability across different growth stages. The correlation coefficient between RA and body weight was r = 0.65, making it the most important predictor, which was consistent with previous studies linking body surface area to livestock body weight [35]. In contrast, contour perimeter (CP, r = 0.45) and ellipse eccentricity (E, r = 0.35) contributed less, suggesting that shape-related features may have limited utility unless integrated with more advanced shape analysis methods.

Compared with traditional manual measurements or simple regression models [36], the combination of EcoSegLite and random forest not only achieved a higher degree of automation and scalability but also exhibited good generalization performance, although its accuracy could still be affected by image quality and segmentation errors. The distribution of standard deviations reflected the natural variability among samples, while the model’s adaptability to such variability indicated its ability to maintain stable performance across different body conformations and postures. Future work could focus on expanding the dataset in both scale and diversity to further enhance generalization capacity and cross-scenario adaptability.

### 4.2. Feeding Optimization and Environmental Benefits

By monitoring pigs’ full-cycle weight and integrating data on feed consumption and manure output, this study successfully implemented precision feeding optimization, significantly reducing resource consumption and environmental impact. Growth curve analysis revealed that feeding strategies guided by real-time weight estimation resulted in an average reduction of 7.8% in total feed consumption and 11.9% in daily manure output. This translates directly into a 5.1% decrease in the carbon footprint per kilogram of pig (from 8.3 kg ${\mathrm{CO}}_{2}$-eq/kg pig to 7.9 kg ${\mathrm{CO}}_{2}$-eq/kg pig, *p* < 0.05), aligning with research findings that precision feeding effectively mitigates resource overuse and waste [37]. Specifically, feed production emissions decreased by 6.5%, primarily due to reduced demand for nitrogen-based fertilizers, while enteric fermentation and manure management emissions declined by 4.2% and 5.3%, respectively, attributable to improved growth efficiency and reduced total manure output. This multifaceted emission reduction contrasts sharply with the traditional uniform feeding approach, which often leads to excessive emissions [38].

The significant reductions of 7.8% in feed intake and 11.9% in manure output result from the dynamic adjustment of precise feed allocation driven by real-time weight estimation. By increasing the corn proportion to 68% and soybean meal to 18–20%, the optimized feeding likely enhanced feed conversion rates, reducing overconsumption. The decrease in manure output may stem from improved metabolic efficiency in pigs and reduced feed waste; combined with nitrogen excretion rate data, we estimate a nitrogen loss reduction of approximately 5–10%, further validating the effectiveness of the 5.3% reduction in manure management emissions. The 5.1% reduction in carbon footprint observed in this study reflects the synergistic emission reduction effects from feed production and enteric fermentation, highlighting the critical role of full-cycle monitoring in facilitating dynamic feeding adjustments.

Although this study achieved significant emission reduction benefits, such as a 7.8% decrease in feed consumption and a 5.1% reduction in carbon footprint, surpassing the typical 3–5% reduction range of conventional sensor-based feeding systems, the results obtained under the controlled conditions of Pianguan Farm, Shanxi, may exhibit context-specificity. The statistical significance (*p* < 0.05) confirmed the efficacy of this approach; however, the 5.1% overall reduction also suggests that future optimizations might target energy use in feed processing or transportation. Future research should focus on enhancing monitoring during the piglet phase through multimodal data, conducting cross-farm validation under more diverse conditions, and quantifying the long-term environmental and economic benefits of precision feeding systems, while also considering the influences of seasonal feed availability and pig health status.

### 4.3. Challenges and Opportunities for Scalable Sustainable Farming

The synergistic application of EcoSegLite segmentation technology and the random forest regression model provided a scalable paradigm for sustainable pig production, capable of effectively reducing resource waste and environmental impact. Although the reduction in carbon footprint was only 5.1%, this figure, while modest, still contributed to global efforts to mitigate the agricultural sector’s 14.5% share of greenhouse gas emissions. Deploying the lightweight model on edge devices improved accessibility for small- and medium-scale farms, thereby promoting the adoption of precision agriculture [39]. However, several challenges remain for large-scale implementation.

At present, the scope of deployment was limited to 63 pigs in the Pianguan farm, with cross-site validation in regions such as Fenyang or Puxian constrained primarily by the time-intensive calibration process required for different breeds and management systems, as well as a shortage of trained personnel. In high-density farming environments, the mean average precision (mAP50) of EcoSegLite decreased by 3.8%, indicating the need to incorporate transfer learning or multi-object tracking techniques to enhance segmentation robustness. The impacts of climate change on camera performance and pig behavior also necessitate the development of cross-regional datasets or adaptive modules. Expanding the sample size and conducting tests in different agroecological zones would help improve the model’s robustness. In parallel, collaborating with agricultural extension services to develop training programs and automated platforms could help alleviate personnel shortages. These technological advancements highlight the transformative potential of computer vision and machine learning in advancing sustainable livestock farming.

## 5. Conclusions

This study optimized the feeding management strategy by developing the EcoSegLite model, successfully achieving the goal of reducing the carbon footprint of pig production. The model validated its effectiveness in segmenting pig back images, with mAP50 at 96.7% and mAP50-95 at 82.3%. Due to its low computational cost of 1.60 M parameters and 4.0 GFLOPs, it significantly outperforms baseline models. The integration of this method with the Random Forest weight estimation model (MAE 3.2 kg, $R^{2}$ 0.92) at the Pianguan farm allowed for optimized feeding, which resulted in a 7.8% reduction in feed intake, an 11.9% reduction in manure output, and a 5.1% reduction in carbon footprint. These achievements, made possible by precise feed adjustments driven by real-time weight estimation and full-lifecycle monitoring data, significantly reduced the carbon footprint and supported the realization of sustainable livestock farming.

While the results are encouraging, their generalizability still requires validation due to the limited sample size of 63 pigs and the specific conditions of the Pianguan farm. Future research should focus on: first, developing adaptive segmentation algorithms to address posture variability and improve accuracy in complex scenarios; second, exploring multi-sensor fusion technologies to further reduce the weight estimation mean absolute error (MAE) to below 2.5 kg; and finally, quantifying the long-term environmental and economic benefits of EcoSegLite through multi-season data and multi-farm trials covering at least 500 pigs over two years. These advancements will enhance the model’s applicability under diverse farm conditions, promoting the sustained reduction of global agricultural emissions.

## Figures and Tables

**Table 1:** Feeding frequency and ratios at different growth stages.

Growth Stage	Weight Range (kg)	Feeding Frequency (Times/Day)	Corn (%)	Soybean Meal (%)	Wheat Bran (%)	Compound Feed and Concentrates (%)
Weaning Period	7.0–15.0	6–8	60	20	5	15
Fattening Stage I	15.1–40.0	3	60	22	10	8
Fattening Stage II	40.1–80.0	3	65	18	13	4
Fattening Stage III	>80.1	3	67	15	14	4

**Table 2:** Feed consumption quantities.

Breed	Feed Consumption Quantities(t)			
	Corn	Soybean Meal	Wheat Bran	Compound and Concentrated Feed
Landrace	35	12	4	3
Jinfen White	26	9	2	2
Duroc	54	27	9	8

**Table 3:** Emission factors for fattening pigs of different breeds.

Breed	Manure Yield (kg/Head·Day)	Nitrogen Emissions (g/Head·Day)	Greenhouse Gases (kg/Head·Day)	Nitrogen Excretion Rate (kg N/1000 kg Pig Mass·Day)
Landrace	3.3	26	0.34	0.55
Jinfen White	3.8	33	0.42	0.58
Duroc	2.8	22	0.3	0.52

**Table 4:** N_2_O direct emission factors for different manure management systems.

System	Emission Factor
Open Lagoon	0
Indoor Storage	0.002
Solid Storage	0.005
Liquid/Slurry (Natural Crust)	0.005
Liquid/Slurry (No Natural Crust)	0
Anaerobic Digester	0
Aerobic Treatment (Natural Vent.)	0.01
Aerobic Treatment (Forced Vent.)	0.005
Composting (Container)	0.006
Composting (Intensive Turned)	0.1
Composting (Passive Stack)	0.01

**Table 5:** Ablation experiment of model components.

YOLO 11	ShuffleNetV2	LDConv	ACmix	mAP (%)		F1-Score (%)	Params	GFLOPs	FPS
				50	50–95				
✓				93.2	76.2	86.6	2.59 M	6.4	99.4
✓	✓			93.3	78.1	87.2	1.71 M	4.1	80.2
✓	✓			94.9	80.2	89.1	2.17 M	5.5	85.2
✓	✓		✓	95.1	79.8	89.4	2.60 M	6.5	103.5
✓	✓	✓		94.8	79.4	88.8	1.60 M	4.0	81.9
✓	✓		✓	95.3	80.5	90.3	1.72 M	4.1	91.0
✓	✓	✓	✓	96.2	81.2	91.4	2.18 M	5.5	93.4
✓	✓	✓	✓	96.7	82.3	92.0	1.60 M	4.0	98.2

Note: All models use YOLO 11 as the baseline. Components are incrementally added to evaluate their impact on performance metrics.

**Table 6:** Comparison results of pig back segmentation with different models.

Model	mAP50	mAP50-95	F1-Score	Params (M)	GFLOPs	FPS
YOLOv8n-seg	92.6	75.6	85.2	2.71	6.9	96.1
YOLOv8s-seg	93.2	76.3	86.1	9.83	23.6	82.8
YOLOv10n-seg	92.4	75.4	85.1	2.76	8.4	93.3
YOLOv10s-seg	93.1	76.0	85.9	8.19	24.8	84.6
YOLO11n-seg	93.2	76.2	86.6	2.59	6.4	99.4
YOLO11s-seg	93.7	77.1	87.3	9.43	21.6	87.3
Mask R-CNN	92.1	74.4	85.3	44.12	25.5	48.0
SOLOv2	89.2	70.5	83.2	46.25	154.5	52.3
YOLACT++	86.5	73.8	85.0	53.72	52.5	75.2
EcoSegLite (ours)	96.7	82.3	92.0	1.60	4.0	98.2

**Table 7:** Statistical summary of features for pig weight estimation.

Variable	Min	Max	Mean	SD	r
Pig Weight (kg)	48.3	121.7	74.9	19.6	–
RA (%)	10.2	49.8	28.4	9.1	0.65
CP (cm)	69.8	189.3	127.6	28.4	0.45
BL (cm)	70.5	132.1	98.7	15.3	0.55
BW (cm)	31.4	79.3	53.9	12.4	0.50
E	0.21	0.79	0.49	0.13	0.35

**Table 8:** Cross-validation results for weight estimation across different farms.

Farm	MAE (kg)	$R^{2}$	MAPE (%)	RMSE (kg)
Pianguan	3.2	0.92	2.4	4.0
Fengyang	3.5	0.91	2.6	4.3
Puxian	3.7	0.89	2.8	4.6

Note: MAE = Mean Absolute Error, MAPE = Mean Absolute Percentage Error, RMSE = Root Mean Square Error. All results are based on 5-fold cross-validation.

## Data Availability

The datasets used in this study are not publicly available due to strict privacy protection requirements and confidentiality agreements with the cooperating third-party pig farm. However, upon reasonable request and with the explicit permission of the pig farm, the relevant data can be obtained from the corresponding author.
